# Osteogenic stimulatory conditions enhance growth and maturation of endothelial cell microvascular networks in culture with mesenchymal stem cells

**DOI:** 10.1177/2041731412443236

**Published:** 2012-04-04

**Authors:** Torbjorn O Pedersen, Anna L Blois, Ying Xue, Zhe Xing, Michele Cottler-Fox, Inge Fristad, Knut N Leknes, James B Lorens, Kamal Mustafa

**Affiliations:** 1 Department of Clinical Dentistry—Center for Clinical Dental Research, University of Bergen, Norway; 2 Department of Biomedicine, University of Bergen, Norway; 3 Department of Pathology—Cell Therapy and Transfusion Medicine, University of Arkansas for Medical Sciences, Little Rock, AR, USA

**Keywords:** endothelial cells, mesenchymal stem cells, microvascular networks, osteogenesis

## Abstract

To optimize culture conditions for in vitro prevascularization of tissue-engineered bone constructs, the development of organotypic blood vessels under osteogenic stimulatory conditions (OM) was investigated. Coculture of endothelial cells and mesenchymal stem cells was used to assess proangiogenic effects of mesenchymal stem cells on endothelial cells. Four different culture conditions were evaluated for their effect on development of microvascular endothelial cell networks. Mineralization, deposition of extracellular matrix, and perivascular gene expression were studied in OM. After 3 days, endothelial cells established elongated capillary-like networks, and upregulated expression of vascular markers was seen. After 15 days, all parameters evaluated were significantly increased for cultures in OM. Mature networks developed in OM presented lumens enveloped by basement membrane-like collagen IV, with obvious mineralization and upregulated perivascular gene expression from mesenchymal stem cells. Our results suggest osteogenic stimulatory conditions to be appropriate for in vitro development of vascularized bone implants for tissue engineering.

## Introduction

In a series of studies focused on developing artificial scaffolding for bone tissue engineering, our group has developed artificial scaffolds and culture conditions adequate to begin translational studies aimed at clinical use in reconstruction of bone defects.^1^ However, the ability to create a functional vasculature in bioengineered tissue is an unresolved challenge in regenerative medicine.^2–4^ In particular, developing an adequate supply of oxygen and nutrients to cells within artificial scaffolds limits the size of defects for which tissue engineering might be a realistic treatment option. In vitro prevascularization, where a vascular bed is developed before constructs are used in reconstructive surgery, has been proposed as a way to overcome this obstacle.^2^

By combining individual vessel components such as endothelial cells (ECs), vascular smooth muscle cells (vSMCs), and basement membrane proteins, several authors have been able to construct a functional vasculature in vivo.^5–7^ Perivascular mural cells have been shown to regulate proliferation of ECs and promote vascular maturation during the development of functional blood vessels.^8^ In order for newly developed blood vessel systems to maintain size, function, and cell survival, endothelial/mural cell connections and subsequent production of basement membrane proteins are needed.^8–10^ The vascular endothelial growth factor (VEGF) and angiopoietin ligand/receptor systems include the most important signaling molecules in development and regulation of blood vessels.^11^ Bone marrow–derived mesenchymal stem cells (MSCs) have shown the ability to support vascular development in the presence of a collagen–fibronectin gel,^12^ and to stimulate vascular ingrowth into collagen sponges.^13^ Differentiation of MSC into vascular cells depends on signals provided by the local environment, in particular the extracellular matrix produced by ECs.^14^ In addition, the influence of ECs on osteogenic differentiation of MSC has been recognized by several authors.^15–17^

Despite extensive efforts to understand coculture systems in general, limited attempts have been made to clarify ideal culture conditions for prevascularization of tissue-engineered bone. As pointed out by Ma et al.,^18^ the majority of coculture studies have focused on osteogenesis or angiogenesis. However, in order to develop prevascularized bone constructs, culture conditions must support both the formation of functional vessels and osteogenesis. The aim of the current study was therefore to examine the mechanisms and the functional formation of endothelial microvascular networks in cocultures of primary human MSC and human umbilical vein ECs. Culture medium enriched with dexamethasone, ascorbic acid, and β-glycerophosphate (DAG) is an established method for inducing osteogenic differentiation of MSC.^19^ We hypothesized that this osteogenic stimulatory medium (OM) would support formation and stabilization of endothelial networks, in addition to osteogenic differentiation and mineralization. Our results show the ability of OM to stimulate endothelial microvascular network development, and to support perivascular and osteogenic differentiation of MSC.

## Materials and methods

### Cells

Human umbilical vein ECs were purchased from Lonza (Clonetics^®^, Walkersville, MD) and expanded in Endothelial Cell Growth Medium 2 (EGM-2^®^) (Lonza). Primary human bone marrow–derived MSCs were purchased from StemCell Technologies (Vancouver, BC) and expanded in MesenCult^®^ (MC) complete medium (StemCell Technologies). Flow cytometry was performed to assess purity of MSC, and >90% of the cells expressed CD29, CD44, CD105, and CD166, while <1% of the cells expressed CD14, CD34, and CD45. Cells from passages 2–6 were used. All cells were cultured at 37°C in a humid atmosphere containing 5% CO_2_.

### Organotypic blood vessel assay

ECs used for live fluorescence imaging were infected with retrovirus carrying a green fluorescent protein (GFP)–expressing construct at an early passage, a procedure described previously.^5,20^ An in vitro organotypic blood vessel system was used,^21^ with MSC used in this study as supporting cells. Briefly, 25,000 MSCs and 5000 ECs were seeded in a half-area 96-well microplate and allowed to form capillary-like networks. The first live fluorescence imaging was made after 72 h. One-fourth of the culture medium was changed for all groups every second day until cells were fixed in 4% paraformaldehyde (PFA) after 15 days. Cells were cultured in EGM-2, MC, OM, and EGM + OM (Table 1). Automated high-throughput imaging was performed to analyze the EC/MSC coculture assay on a BD Pathway 855 bioimaging system (BD Bioscences, San Jose, CA). The 2 × 2 montages of each well were acquired with a 10× lens. Noise reduction (rolling ball) and adjustment of image threshold were performed with AttoVision v1.6.1 software (BD Bioscences). The threshold for minimum segment size in the networks was set to 5000 pixels.

**Table 1.** Media used for coculture experiments

Full name	Abbreviation	Supplements
Endothelial growth medium-2	EGM	10% FBS, 0.1% hEGF, 0.1% hydrocortisone, and 0.1% GA-1000
MesenCult	MC	MesenCult mesenchymal stem cell stimulatory supplements
Osteogenic medium	OM	15% MesenCult osteogenic stimulatory supplements
		50 µg/mL ascorbic acid
		10^−8^ M dexamethasone
		3.5 mM β-glycerophosphate
Endothelial growth medium-2 + osteogenic medium	EGM + OM	As for EGM < 7 days and OM > 7 days

FBS: fetal bovine serum; hEGF: human epidermal growth factor.

### Coculture of MSC/EC

To evaluate EC gene expression, EC and MSC were cultured in six-well plates (NUNC, Roskilde, Denmark) at a ratio of 5:1 (10^5^ and 2 × 10^4^ cells, respectively) or EC alone (10^5^ cells) under the same conditions for 3 days. EGM-2 culture medium was used. After 3 days, cells were trypsinized, and positive isolation of EC was performed with CD31 Endothelial Cell Dynabeads^®^ (Invitrogen, Carlsbad, CA) according to the manufacturer’s instructions. To evaluate the expression of VEGFa, indirect coculture was performed with cell culture inserts (NUNC) at a pore size of 3 µm. ECs were centrifuged and frozen in liquid nitrogen before RNA isolation.

To evaluate stem cell differentiation, MSCs were cocultured with EC in six-well plates at a ratio of 5:1 (10^5^ and 2 × 10^4^ cells, respectively) or MSC alone (10^5^ cells) under the same conditions for 15 days. Cells were cultured in the following culture media: EGM-2 (Lonza), osteogenic medium (OM) (StemCell Technologies), and EGM + OM (Table 1). Culture media were changed every 3 days. After 15 days, cells were trypsinized and EC depleted with CD31 Endothelial Cell Dynabeads according to the manufacturer’s instructions. MSCs were centrifuged and frozen in liquid nitrogen before RNA isolation.

### Real-time reverse transcriptase polymerase chain reaction

RNA was isolated and real-time reverse transcription–polymerase chain reaction (RT-PCR) was performed as previously described.^22^ At 3 days, Taqman^®^ gene expression assays (Applied Biosystems™) were used to detect mRNA levels of glyceraldehyde-3-phosphate dehydrogenase (GAPDH), angiopoietin 1 (Ang1), angiopoietin 2 (Ang2), von Willebrand factor (vWF), and VEGFa from EC. Assays for GAPDH, SM22-α, and α-smooth muscle actin (α-SMA) were used to detect mRNA levels in MSC after 15 days of culture in OM. The data were analyzed with a comparative Ct method, where expression levels of the genes were normalized to the HouseKeeper index and GAPDH served as endogenous control.

### Histological staining

Extracellular matrix staining of collagen IV was performed on fixed cocultures.^21^ Briefly, monoclonal mouse anticollagen IV antibody (Millipore, Billerica, MA) diluted 1:200 in phosphate-buffered saline (PBS)/2% fetal bovine serum (FBS) and Alexa546-conjugated goat antimouse IgG secondary antibody diluted 1:3000 in PBS/2% FBS (Invitrogen) were used. Images were acquired with a Zeiss LSM 510 Meta confocal microscope (Carl Zeiss, Oberkocken, Germany). To identify calcium, alizarin red S staining was performed. Two percent of alizarin red S powder (Sigma-Aldrich, St. Louis, MO) was dissolved in distilled water, and pH was adjusted to 4.2 with 0.5% ammonium hydroxide. Cocultures were stained for 3 min and imaged with a Nikon TS100 microscope (Nikon, Tokyo, Japan). ECs were stained with TRITC–UEA 1 lectin (Sigma-Aldrich) (diluted 1:1000) for 45 min in room temperature protected from light. MSCs were stained with mouse antihuman α-SMA (Santa Cruz Biotechnology, Santa Cruz, CA) diluted 1:200 in PBS/2% FBS, and with Alexa594-conjugated goat antimouse IgG secondary antibody diluted 1:3000. 4′,6-Diamidino-2-phenylindole (DAPI) staining was done in 1:3000 dilution for 2 min in room temperature.

### Statistical analysis

Statistical evaluation of total tube length, total tube area, and total tube perimeter was acquired through the “tube formation” analysis module in AttoVision v1.6.1. Tube total length was defined as the total number of pixels comprising the network in the image field. Calculations of tube total area and perimeter treat a single particle as an aggregate of square pixels, where a single-pixel segment has an area of 1 and a perimeter of 4, leading to consistent results across all scales of magnification. PCR data presented are from at least three parallel samples and were repeated with different stem cell donors to confirm consistency. SPSS Statistics 19.0 (IBM, Armonk, NY) was applied for statistical processing and analysis, and groups were compared with the independent samples t-test. The significance level was set to p < 0.05.

## Results

### Endothelial microvascular networks after coculture of ECs and bone marrow–derived MSCs

**Figure 1.**
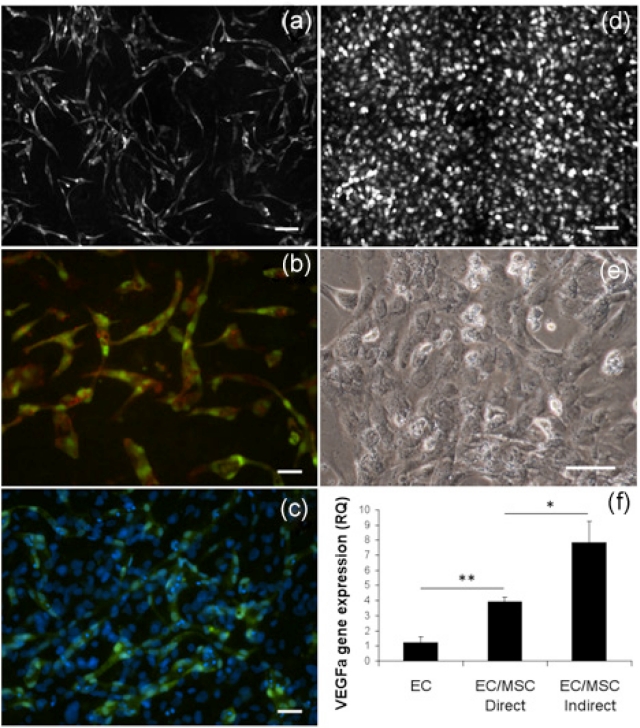
Endothelial network establishment in coculture with bone marrow–derived MSCs. (a) The 2 × 2 montage image (10×) with live fluorescence microscopy from network of GFP-expressing EC cocultured with MSC. At 6 days, ECs have organized into stabilized networks. Scale bar = 100 µm. (b) UEA lectin staining of fixed GFP-expressing EC (20×) organized in elongated structures at 6 days. Scale bar = 50 µm. (c) DAPI staining (20×) showing the confluent layer of MSC supporting EC network formation at 6 days. Scale bar = 50 µm. (d) Live fluorescence microscopy after 6 days of monocultured GFP-expressing EC (10×) that did not spontaneously organize into capillary-like networks. Scale bar = 100 µm. (e) Monocultured EC (20×) presented a cobblestone-like morphology. Scale bar = 50 µm. (f) EC expression of VEGFa was significantly upregulated in coculture at 3 days, but also significantly lower than in indirect culture with shared culture medium. *p < 0.05, **p < 0.01. GFP: green fluorescent protein; EC: endothelial cell; MSC: mesenchymal stem cell; DAPI: 4′,6-diamidino-2-phenylindole; VEGF: vascular endothelial growth factor.

Development of capillary-like structures in EC/MSC coculture followed the same pattern as the EC/vSMC coculture,^21^ with EC forming an interconnected network over a confluent layer of MSC (Figure 1(c)). In coculture, branching of EC happened within days, and a well-established network was observed at day 6 (Figure 1(a)). In monocultured EC, endothelial networks could not be observed at day 6 (Figure 1(d)), with EC presenting cobblestone-like morphology (Figure 1(e)), illustrating the importance of supporting cells for EC in the process of network formation. At 3 days, mRNA expression of VEGFa was significantly upregulated in coculture compared to EC alone (p < 0.01), but also significantly lower compared to the indirect culture system (p < 0.05) (Figure 1(f)).

### Endothelial gene expression in coculture with bone marrow–derived MSCs

**Figure 2.**
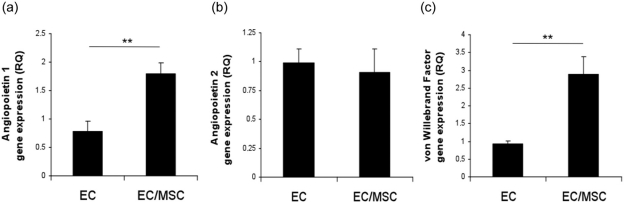
Endothelial gene expression in coculture at 3 days. Expression of (a) angiopoietin 1, (b) angiopoietin 2, and (c) von Willebrand factor in monoculture and coculture. **p < 0.01. EC: endothelial cell; MSC: mesenchymal stem cell.

Gene expression of EC markers Ang1, Ang2, and vWF was evaluated at 3 days (Figure 2). mRNA expression of Ang1 and vWF was significantly upregulated in coculture compared to monoculture (p < 0.01), whereas expression of Ang2 was not significantly different in the two culture systems.

### Effect of osteogenic stimulatory medium on growth and maturation of endothelial microvascular networks

**Figure 3.**
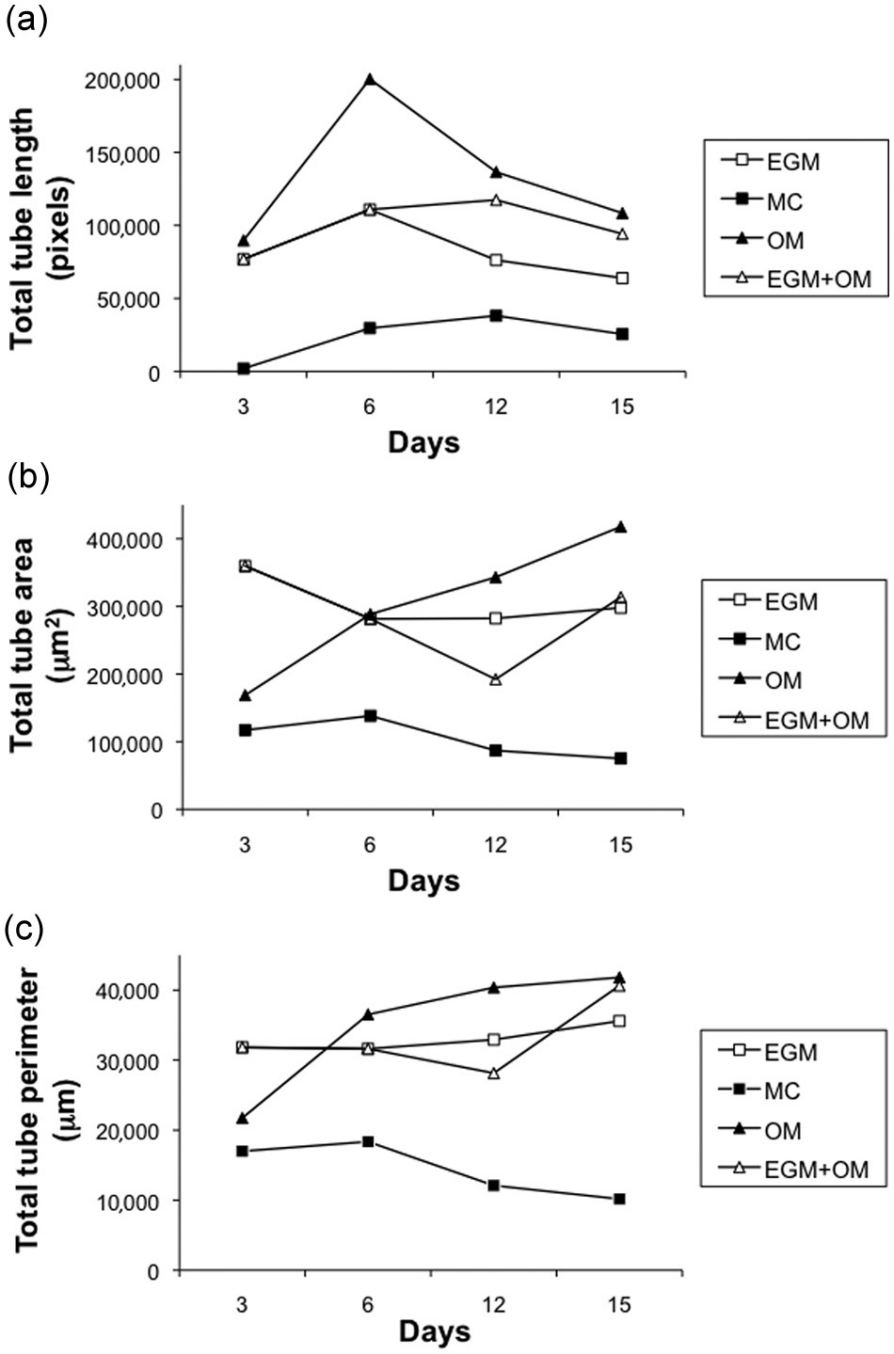
Growth and maturation of endothelial networks in four culture conditions. (a) Total tube length was increased in OM from establishment of networks at 3 days and for the remaining experimental period. For all groups, the length decreased as networks stabilized. (b) Total tube area was greater in networks generated in EGM at 3 days. At 6 days, networks generated in OM presented the greatest total tube area. Networks generated in EGM but maintained in OM after 7 days saw a decreased area after OM was added, followed by a steady increase. A similar tendency could be observed for (c) total tube perimeter. Results show that OM initially stimulates network proliferation, and subsequently promotes development of stable networks with higher length, area, and perimeter. EGM: Endothelial Cell Growth Medium; MC: MesenCult.

**Figure 4.**
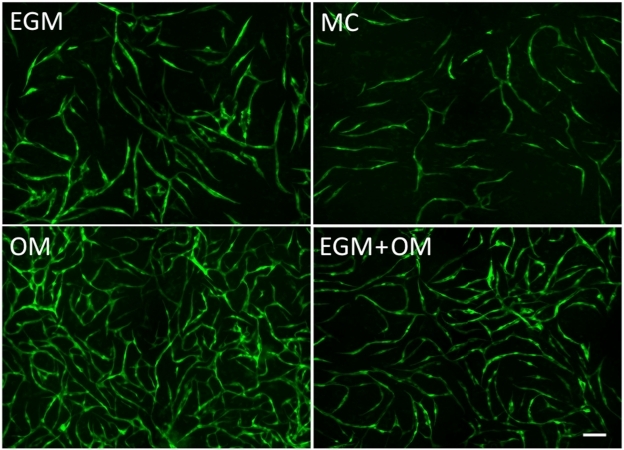
Established endothelial networks after coculture in different culture media. The 2 × 2 montage live fluorescence microscopy of GFP-expressing EC (10×) under four culture conditions after 15 days. (a) EGM, (b) MC, (c) OM, and (d) EGM + OM. Seeded EC/MSC are the same for all four conditions. Scale bar = 100 µm. EGM: Endothelial Cell Growth Medium; EC: endothelial cell; MC: MesenCult.

**Figure 5.**
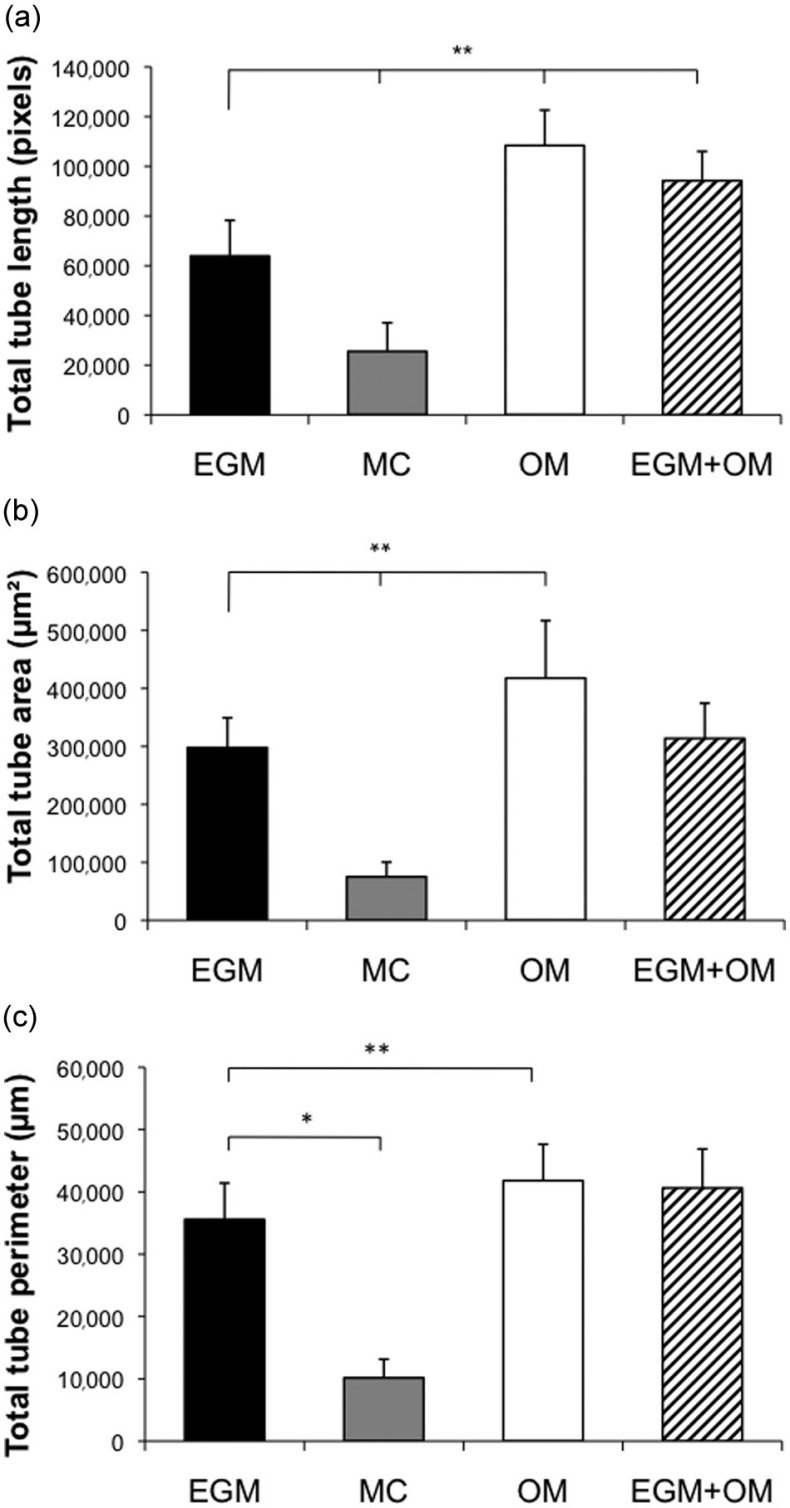
Network evaluation parameters at 15 days. Quantifications of (a) total tube length, (b) total tube area, and (c) total tube perimeter after 15 days coculture in four different culture media. Statistical differences indicated are compared to coculture in EGM. * p < 0.05, **p < 0.01. EGM: Endothelial Cell Growth Medium; EC: endothelial cell; MC: MesenCult.

Quantitative measurements of total tube length in coculture showed the highest values under osteogenic stimulatory conditions (Figure 3(a)), indicating increased cell proliferation of EC during the initial phase. Analysis of total tube area and perimeter were performed to evaluate network maturation (Figure 3(b) and (c)), and at 3 days, both parameters were lower in OM compared to EGM. At 6 days, measurements were similar under both culture conditions and increasingly higher in OM until cocultures were fixed at 15 days (Table 2). A similar trend was observed in cocultures where EGM medium was used for the first 7 days and osteogenic medium from days 7 to 15. An increase in total tube length as well as decreased total tube area and perimeter were seen after the change to osteogenic medium. At day 15, the EGM + OM networks were stable with a higher total length than the EGM group, but lower than the OM group, while the total tube area and perimeter surpassed the EGM group (Figure 3(a) to (c)). Endothelial microvascular networks developed in OM had the most elaborate interconnections and structures after 15 days (Figure 4), as well as significantly increased network growth and maturation (p < 0.01) (Figure 5).

**Table 2.** Quantitative assessment of endothelial microvascular networks

		3 days	6 days	12 days	15 days
Total tube length (pixels)					
EGM	Mean	76,903	110,871	76,217	63,936
	SD	21,807	37,000	22,337	14,332
MC	Mean	1942	29,629	38,141	25,522
	SD	639	23,495	12,189	11,506
OM	Mean	89,719	200,287	136,547	108,389
	SD	38,680	44,099	18,358	14,228
EGM + OM	Mean	76,900	110,870	117,420	94,210
	SD	21,807	37,000	29,710	11,790
Total tube area (µm^2^)					
EGM	Mean	359,761	281,606	282,345	297,982
	SD	44,275	49,583	50,747	51,328
MC	Mean	117,142	138,190	86,915	75,141
	SD	53,779	55,923	31,000	25,226
OM	Mean	168,686	288,537	343,105	417,791
	SD	50,828	48,098	96,469	99,061
EGM + OM	Mean	359,761	281,606	192,308	313,728
	SD	44,275	49,583	55,478	60,785
Total tube perimeter (µm)					
EGM	Mean	31,824	31,619	32,920	35,584
	SD	4246	6270	4044	5840
MC	Mean	16,996	18,362	12,095	10,165
	SD	9407	6212	3607	2878
OM	Mean	21,736	36,516	40,377	41,813
	SD	3846	5358	6350	5836
EGM + OM	Mean	31,824	31,619	28,139	40,629
	SD	4246	6671	7918	6247

EGM: Endothelial Cell Growth Medium; MC: MesenCult; SD: standard deviation.

### Osteogenic stimulatory medium supports perivascular and osteogenic differentiation of MSCs

**Figure 6.**
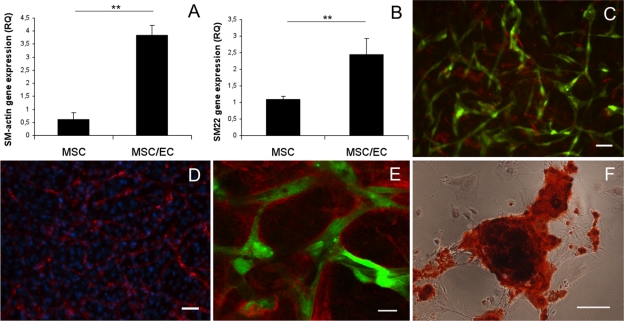
Perivascular gene expression, collagen IV deposition and mineralization in osteogenic stimulatory medium at 15 days. Relative gene expression of (a) α-SMA and (b) SM22-α in monoculture MSC and coculture MSC/EC. **p < 0.01. (c) GFP-expressing endothelial tubes with a representative number of MSC positive for α-SMA (10×). Scale bar = 50 µm. (d) DAPI staining (10×) illustrates the total number of MSC in the network at 15 days. EC are stained with UEA lectin. Scale bar = 50 µm. (e) Representative confocal micrograph (40×) of collagen IV enveloping GFP-expressing endothelial tubes after 15 days. Scale bar = 20 µm. (f) Representative light micrograph (20×) of MSC stained with alizarin red S showing calcium deposits after 15 days. Scale bar = 50 µm. MSC: mesenchymal stem cell; EC: endothelial cell; GFP: green fluorescent protein; DAPI: 4′,6-diamidino-2-phenylindole.

In order to address perivascular differentiation of MSC in osteogenic medium, gene expression of smooth muscle markers after 15 days of coculture was analyzed with real-time RT-PCR. Relative expression of SM22-α and α-SMA was significantly higher (p < 0.01) in coculture compared to monoculture of MSC (Figure 6(a) and (b)). MSC positive for α-SMA were observed through histological staining (Figure 6(c)) and could be compared to the total number of MSC at 15 days culture in OM (Figure 6(d)). Extracellular matrix staining for collagen IV revealed abundant amounts surrounding endothelial tubes (Figure 6(e)). Production of basement membrane proteins indicates a mature vascular network, associated with arrested EC proliferation. Alizarin red S staining showed calcium deposition from differentiated MSC (Figure 6(f)), illustrating the ability of the stem cell population to undergo both perivascular and osteogenic differentiation under the same culture conditions.

## Discussion

A variety of culture systems have been used to investigate crosstalk between ECs and perivascular mural cells.^23^ In order to address vascular development through quantitative high-content imaging techniques, Evensen et al.^21,24^ adapted coculture of EC and vSMCs so as to enable high-throughput screening of EC behavior. Limited information exists to date regarding the functional and molecular mechanisms behind vascular development under osteogenic stimulatory conditions. In the present study, we used coculture of EC and MSC with this novel in vitro angiogenesis screening method to investigate the development of endothelial microvascular networks under different culture conditions, in order to identify a system, which would optimize prevascularization of tissue-engineered bone constructs.

EC recruitment of pericyte precursors and subsequent differentiation during vascular development was investigated by Hirschi et al.,^25^ where smooth muscle markers, SM-myosin, SM22-α, and calponin, were expressed from precursor cells both in vitro and in vivo after coculture with EC. Au et al.^12^ found that in addition to expressing smooth muscle markers in vitro, MSC could support a lasting vasculature in vivo when provided with basement membrane proteins. MSCs have thus been identified as an appropriate cell type for investigating perivascular cell differentiation as well as vascular engineering. In the presence of MSC, EC spontaneously organized into elongated interconnected structures, leading to establishment of endothelial microvascular networks after 3 days. Cellular interactions in coculture have been shown to result in the production of matrix proteins like fibronectin and collagen I and a periendothelial matrix that leads to migratory and morphogenic EC.^21^ Such characteristics were not observed in monoculture in the present study, suggesting that EC may depend on perivascular cells for rapid network organization in vitro.

Several authors have discussed the role of Ang1 as a mediator of vascular maturation, recruiting perivascular cells and maintaining interactions between cells and the extracellular matrix.^26–28^ Vascular development is initiated by VEGF-promoting proliferation, migration and sprouting of ECs,^29,30^ whereas establishment of functional vessels is facilitated by Ang1.^31,32^ Thurston et al.^33^ identified the role of Ang1 in promoting vessels resistant to leakage, compared to vessels solely induced by VEGF. Endothelial microvascular networks were morphologically obvious after 3 days in our study, and real-time RT-PCR analysis was performed in order to better understand the underlying mechanisms on the molecular level. We found significantly higher expression of Ang1 from EC in coculture (p < 0.01).

In quiescent vasculature, expression of Ang2 is barely detectable, but significant upregulation can be observed during angiogenic sprouting.^31^ Angiopoietins 1 and 2 both bind to the Tie2 receptor, and the role of Ang2 in vascular destabilization is mediated through competitive receptor binding with Ang1.^31,34^ The similar expression of Ang2 in both culture systems here indicates established quiescent EC after 3 days, suggesting that the process of network maturation involves interplay between angiopoietins 1 and 2, more specifically through upregulation of Ang1.

The function of VEGF and its receptors in vascular development and angiogenesis has been thoroughly investigated, and their regulatory function in the cardiovascular system was reviewed by Olsson et al.^35^ VEGF is required for development of EC networks in several angiogenesis assays, including coculture with vSMCs and when cultured on a supporting matrix.^21,36^ In agreement with this, we found that expression of VEGF from EC was significantly higher in the cocultures. Increased release of VEGF from MSC through paracrine signaling has previously been reported,^37^ and we report a similar finding of VEGF expression from EC where direct contact yielded significantly lower mRNA compared to the indirect system (p < 0.05). Both in embryonic development and in the formation of blood vessels in adults, it is evident that VEGF and Ang1 work together in a complementary fashion. Our data indicate a combined paracrine and direct contact communication also for tissue-engineered vessels.

vWF is a platelet adhesion mediator known for its essential role in hemostasis, but more recently it has been shown to have multiple roles in vascular development.^38,39^ Enhancement of in vitro angiogenesis through a VEGF receptor 2 (VEGFR-2)–dependent pathway has been achieved through vWF inhibition and is associated with increased release of Ang2.^39^ The same authors also found increased VEGF-induced proliferation after vWF inhibition and attributed the effect to intracellular vWF. Our results suggest a role for vWF in EC stabilization, in that it is associated with loss of proliferation, and shows the interplay of VEGF and angiopoietins in formation of mature endothelial networks. Future studies are warranted to explore the initial molecular mechanisms of endothelial organization in coculture beyond the requirement of VEGF.

Microvascular network formation occurs rapidly following osteogenic stimulation, with OM seeming to exert an initial proliferative effect on EC. β-Glycerophosphate is added to osteogenic medium to mediate mineral formation through hydrolysis by bone cells,^40^ whereas ascorbic acid (AA) is an important cofactor in the synthesis of collagen^41^ and subsequently for the production of osteoid from MSC/osteoblasts (collagen I) and a vascular basement membrane from EC (collagen IV). Proliferation of MSC is influenced by the concentration of AA in culture, with increasing cell proliferation at low concentrations.^41^ In addition to collagen synthesis, AA is required for osteogenic differentiation of MSC in vitro through both enhanced expression of osteoblastic markers and mineralization.^19^ AA also affects EC, with Yue et al.^42^ reporting morphological changes and decreased cellular proliferation of retinal EC when cultured with 75 g/mL AA in vitro. ECs have the ability to take up AA intracellularly and resecrete it into the culture medium,^43^ potentially affecting cocultured MSC. However, studying isolated effects in monocultures is not sufficient to draw conclusions in coculture systems that involve complex interplay between both cell types.

Dexamethasone is used to induce MSC proliferation and osteogenic differentiation, but has also been reported to preserve stem cell characteristics when administered in low dose,^44^ and to reduce total cell number in cultures of bone marrow stromal cells.^45^ Jaiswal et al.^19^ concluded that this paradoxical effect of dexamethasone could be attributed to dosage, duration as well as the stage of differentiation. Measurements of total tube length in the pure OM group were significantly increased compared to the EGM group after 3 days, and continued to be so throughout the experimental period. Indeed, the same trend was seen in the EGM + OM group, where OM was introduced at day 7. This shows the ability of mature microvascular networks to react to stimulation with osteogenic medium, and reenter the proliferative phase. Network maturation parameters, however, were initially lower in OM, a trend also observed in the EGM + OM group. Lian and Stein^46^ determined that growth and differentiation are functionally related in a reciprocal manner, where arrested cell division is needed for differentiation. Delayed network maturation is therefore to be expected in the proliferative phase, and further measurements of total tube area and perimeter in fact showed a linear increase surpassing the EGM group before stabilizing on a significantly higher level. By day 6, all three parameters were highest in OM suggesting that prevascularization of 6 days or longer will benefit from osteogenic stimulatory conditions.

Plasticity of MSC is essential for providing perivascular support and osteogenic differentiation within a tissue-engineered construct. Recruitment of pericytes, cells positive for α-SMA, is a critical step in vascular maturation in vivo, and strongly associated with a stable vascular plexus.^10^ SM22-α is a protein associated with the contractile apparatus of the cytoskeleton and restricted to the smooth muscle cell lineage,^47^ whereas α-SMA is a commonly used marker for the smooth muscle phenotype and highly expressed in quiescent smooth muscle cells. α-SMA is not exclusively expressed in smooth muscle cells, and the findings of α-SMA in MSC are among increasing evidence that pericytes might be MSC residing in close proximity to the microvasculature.^48^ Elevated gene expression of SM22-α and α-SMA from MSC when cocultured with EC shows the ability of MSC to take on a perivascular function in endothelial microvascular networks under osteogenic stimulatory conditions. Further maturation of these networks was demonstrated through extracellular matrix staining, showing tubes enveloped with collagen IV forming basement membrane-like structures, a crucial event in stabilizing newly formed vessels.^8^ Importantly, endothelial tubes presented distinct lumens in a manner comparable to a vascular plexus.

The EC as a mediator of osteogenic differentiation has received much attention,^15,17^ and ECs have been shown to enhance in vivo bone formation when cocultured with MSCs.^22,49^ We investigated if MSCs could serve a perivascular function and differentiate into osteoblasts within the same culture system. Alizarin red S staining of calcium showed obvious positive staining in cocultures after 15 days. This result is in agreement with Ma et al.^18^ who reported that a higher ratio of EC in coculture with MSC resulted in increased calcium deposition, concluding that both stimulation with osteogenic constituents and interactions with EC were necessary for osteogenic differentiation. It has been reported that coculture systems increase the life span and survival of ECs,^50^ a process possibly depending on the coculture ratio, where <50% ECs are reported as a prerequisite for survival in OM.^18^ The relatively high initial percentage (80%) of MSC used in culture in the current study should be considered since we have shown that the MSC population has the ability for both perivascular and osteogenic differentiation. It remains to be determined whether the ECs or the osteogenic medium is the major contributing factor for mineralization in coculture at this ratio. The relative number of the cells in coculture systems is dynamic, where the percentage of EC has been shown to decrease over time.^51^ This supports the notion that MSC facilitates network maturation with subsequent reduced proliferation. The cell ratio used in the current experiments might therefore not be as favorable in other culture conditions; nevertheless, data suggest the high percentage of MSC to be beneficial for endothelial microvascular network development and for the dual function of MSC in OM.

## Conclusion

Under coculture conditions, MSC supported the formation of stable and mature endothelial microvascular networks associated with upregulated expression of Ang1, VEGFa, and vWF. In cultures lasting longer than 6 days, OM enhanced growth and maturation of endothelial microvascular networks. In addition, OM had the ability to support perivascular and osteogenic differentiation of MSC. Future studies are warranted to evaluate vascular development in OM using artificial three-dimensional scaffolds, and our findings suggest that OM would be an excellent culture medium for prevascularization of bone implants.
